# Supplementary material for: Deep topographic proteomics of a human brain tumour
Source: Nat Commun. 2023 Nov 24;14:7710. doi: 10.1038/s41467-023-43520-8 (PMC10673928; doi:10.1038/s41467-023-43520-8)
Supplement: Supplementary file 1 — Supplementary Information [file 41467_2023_43520_MOESM1_ESM.pdf]

Supplementary Information

Deep topographic proteomics of a human brain tumour

Simon Davis[1,2], Connor Scott[3], Janina Oetjen[4], Philip D Charles[1,5], Benedikt M Kessler[1,2], Olaf Ansorge[3], Roman Fischer[1,2] *

[1] Target Discovery Institute, Centre for Medicines Discovery, Nuffield Department of Medicine, University of Oxford, Roosevelt Drive, Oxford, OX3 7FZ, UK

[2] Chinese Academy for Medical Sciences Oxford Institute, Nuffield Department of Medicine, University of Oxford, Roosevelt Drive, Oxford, OX3 7FZ, UK

[3] Academic Unit of Neuropathology, Nuffield Department of Clinical Neurosciences, University of Oxford, John Radcliffe Hospital, Oxford, OX3 9DU, UK

[4] Bruker Daltonics GmbH & Co. KG, Fahrenheitstraße 4, 28359 Bremen, Germany

[5] Big Data Institute, Nuffield Department of Medicine, University of Oxford, Roosevelt Drive, Oxford, OX3 7FZ, UK

* Corresponding author. E-mail: roman.fischer@ndm.ox.ac.uk. Phone: +44 1865 743639

Supplementary Figures

A B C

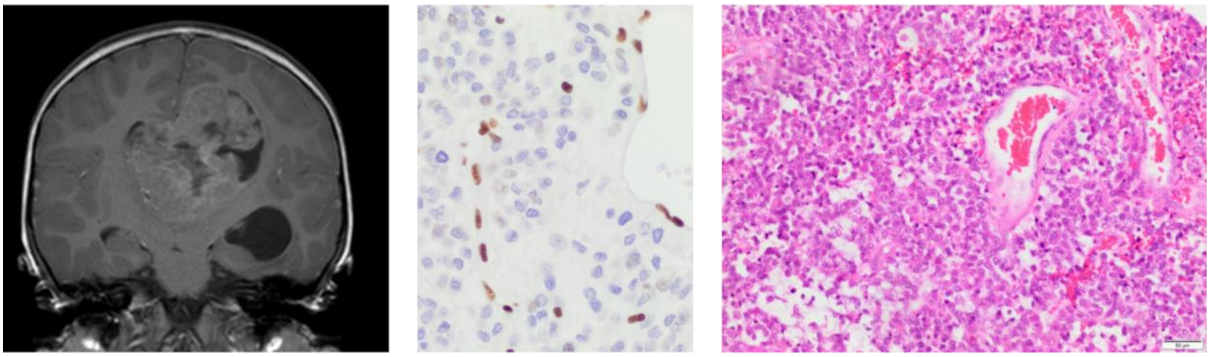

Figure S1 – Clinicopathological data of AT/RT case

(A) Pre-operative MRI T1 with gadolinium in a female patient. (B) SMARCB1 immunohistochemistry staining on biopsy tissue, showing negative staining in tumour cells and retained nuclear expression in host-derived endothelial cells (brown reaction product); lumen of a blood vessel top right. (C) H&E stain of postmortem tissue block showing relative homogeneity and medium-sized tumour blood vessels.

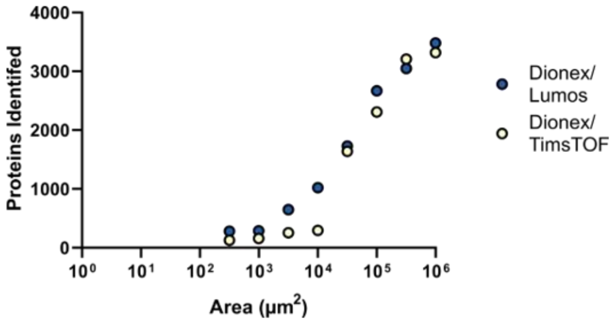

	Side Length (μm)	Area (μm²)	cells
$10^{2.5}$	18	316	2.7
10^{3}	32	1,000	8.6
$10^{3.5}$	56	3,162	27
10^{4}	100	10,000	86
$10^{4.5}$	178	31,623	270
10^{5}	316	100,000	860
$10^{5.5}$	562	316,228	2700
10^{6}	1000	1,000,000	8600

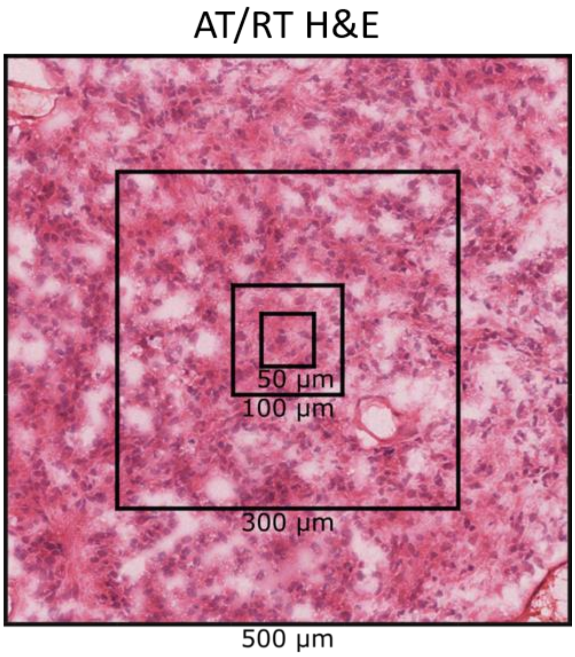

Figure S2 – Tissue area titration across two LC-MS/MS systems
The number of proteins identified from a titration of AT/RT tissue area on two LC-MS/MS systems. Source data are provided as a Source Data file.

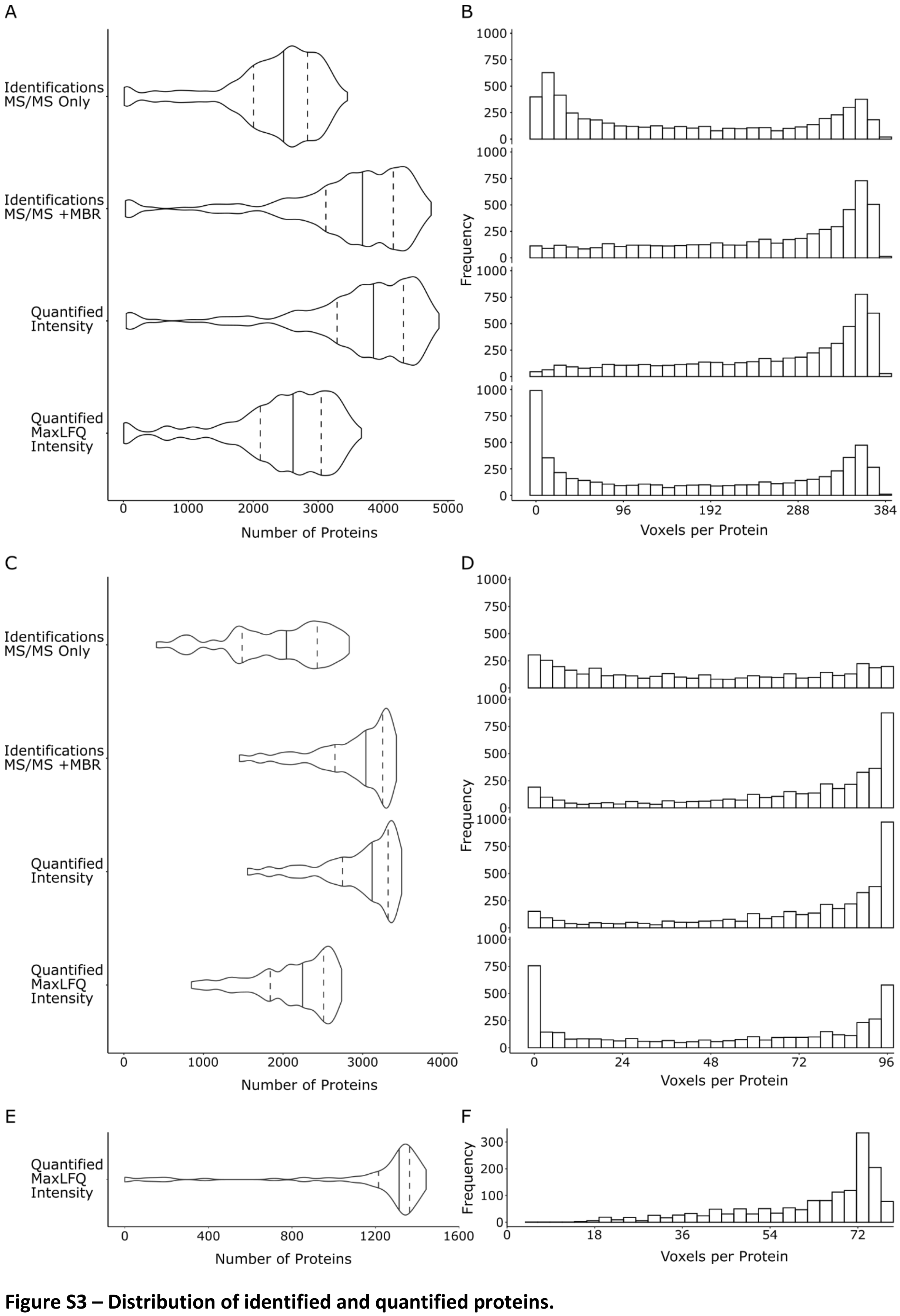

Figure S3 – Distribution of identified and quantified proteins.

(A) 833 µm resolution data. Violin plots showing distributions of the number of identified and quantified proteins per voxel. Solid vertical lines represent the median value. Dashed vertical lines represent upper and lower quartiles. (B) 833 µm resolution data. Histogram showing the number of voxels where each protein was identified/quantified as per panel (A). (C) 350 µm data as in panel (A). (D) 350 µm data as in panel (B). (E) 40 resolution µm data. Violin plot shows distribution of the number of quantified proteins per voxel. Solid vertical line represents the median value. Dashed vertical lines represent upper and lower quartiles. (F) 40 µm resolution data. Histogram showing the number of voxels where each protein was quantified. Source data are provided as a Source Data file.

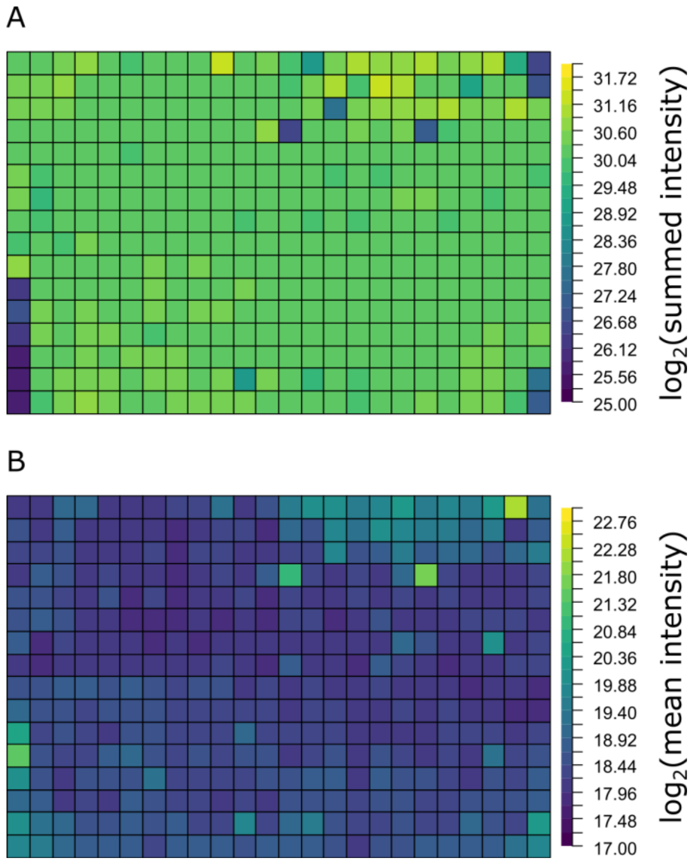

Figure S4 – Aggregate intensity distributions at 833 µm spatial resolution
Maps of the log_2 transformed (A) summed and (B) mean intensities of each voxel.

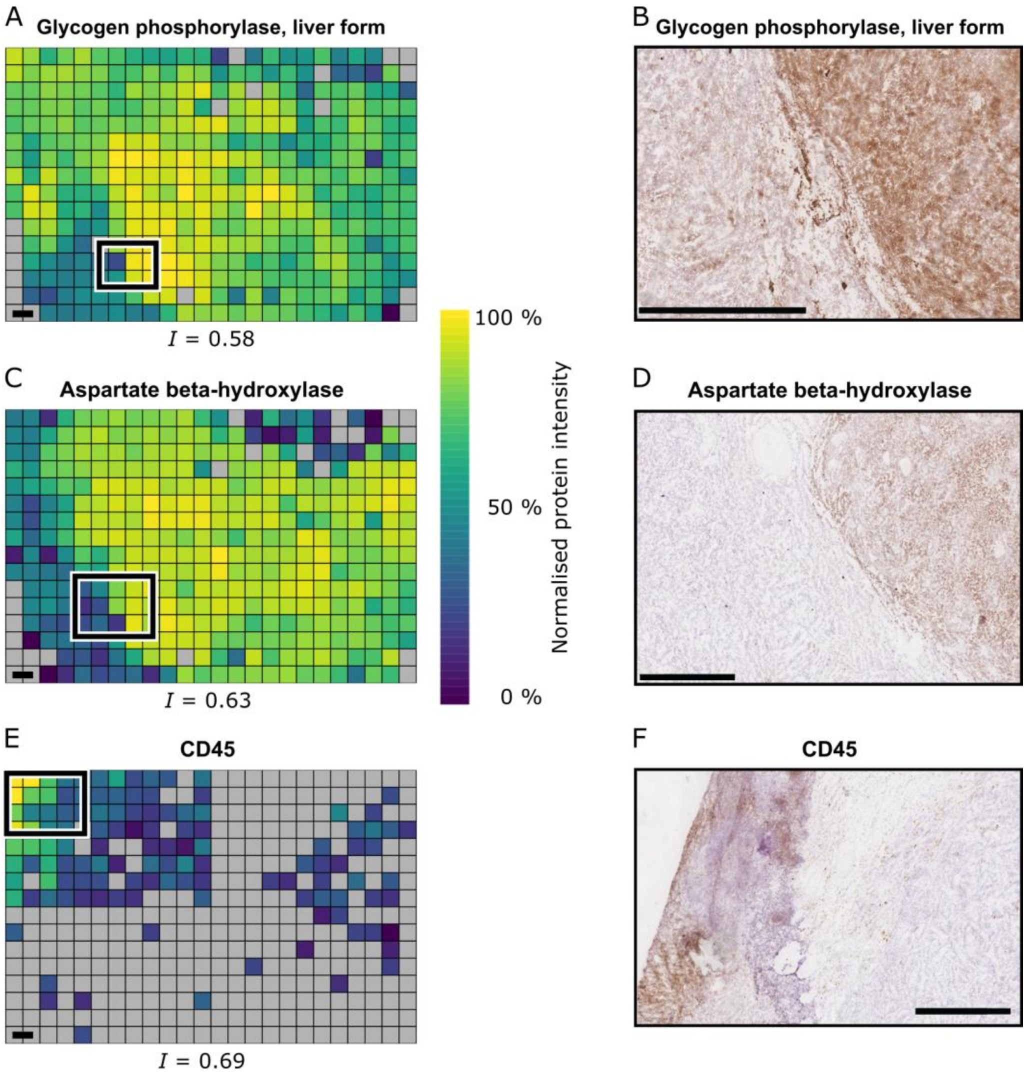

Figure S5 - Immunohistochemistry validation of AT/RT proteomic maps

Proteomic maps of proteins targeted for follow-up IHC staining (n = 1 per protein) and IHC images (A,C,E) Normalised protein intensity maps with their corresponding Moran's Index of spatial autocorrelation (*I*). Normalised protein intensities are scaled separately for each protein. Grey = not detected. Rectangles depict the approximate location displayed in IHC images. (B,D,F) AT/RT tissue stained and visualised by IHC. All scale bars = 1 mm. Full images of the slides are contained in supplementary data file 5.

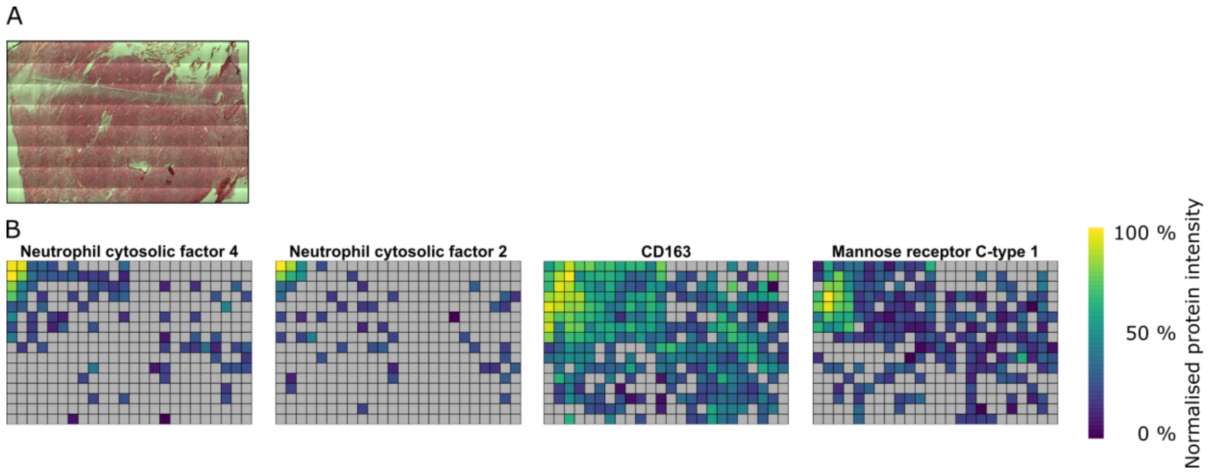

Figure S6 – Proteomic maps of immune cell markers at 833µm spatial resolution

H&E Stained Image of AT/RT Tumour (A). Proteomic maps of immune cell-marker proteins at 833 µm resolution (B). Normalised protein intensities are scaled separately for each protein. Grey = not detected.

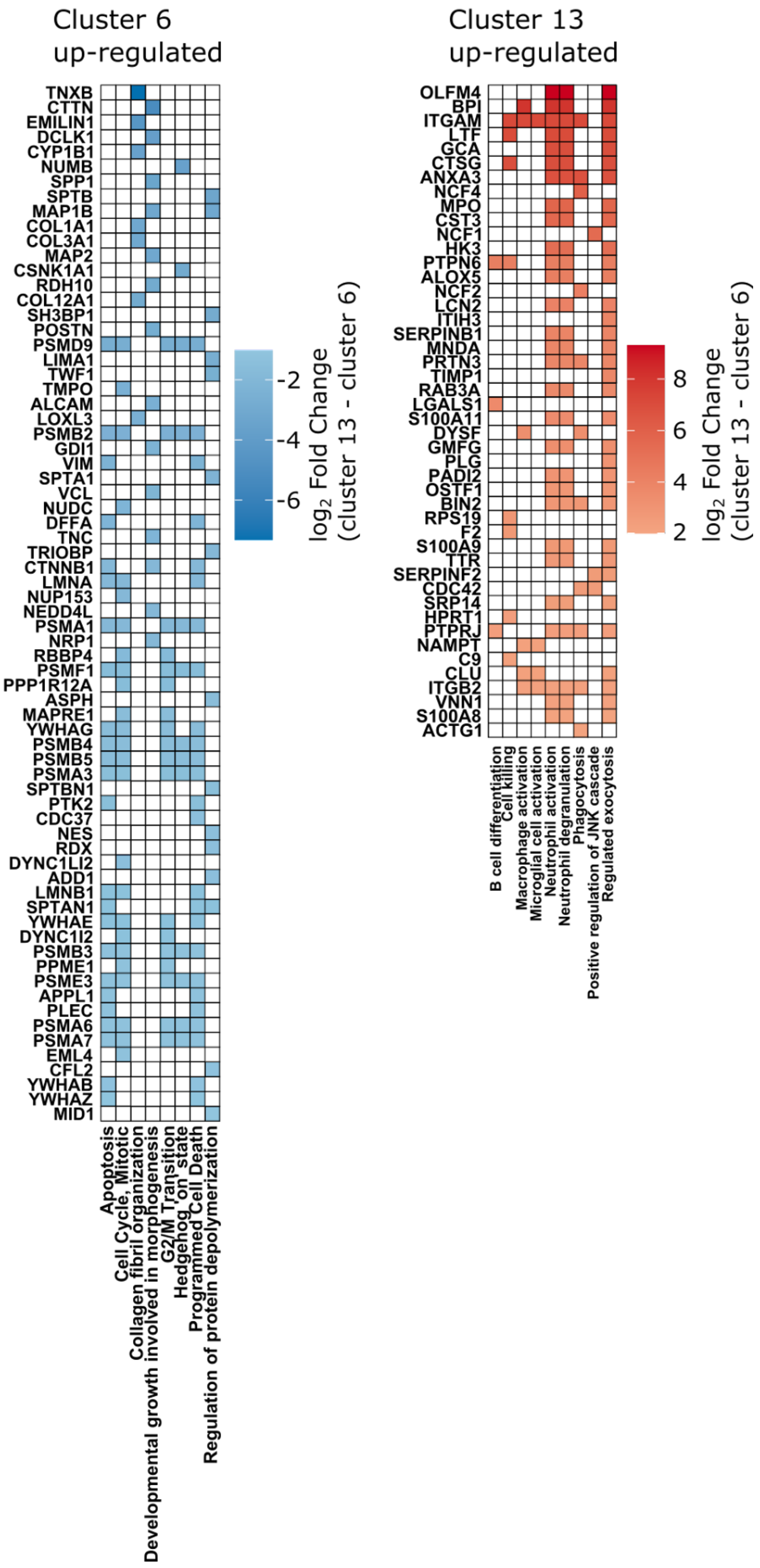

Figure S7 – Pathway enrichment in immune-cell clusters at 833 µm spatial resolution
GSEA of clusters 6 & 13 from Figure 4A. Gene set membership is indicated by colouring the cell with that protein's log2 fold-change.

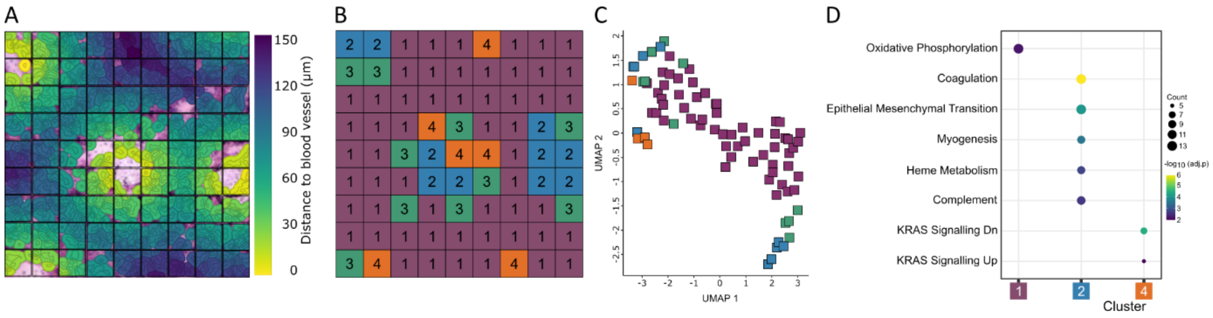

Figure S8 - Dimensionality reduction and gene-set enrichment at 40 µm spatial resolution
(A) Measurement map of distance from each cell to the nearest blood vessel, reproduced from Figure 5B for reference. (B) Map of cluster assignment based on hierarchical clustering and the dynamic tree cut algorithm (spatially unaware), reproduced from figure 5D for reference. (C) UMAP embedding of data coloured by cluster assignment in (B). (D) Enriched MSigDB Hallmark gene sets within marker proteins (two-sided Wilcox test, Benjamini-Hochberg multiple testing correction threshold of 1 %) of clusters shown in the cluster map. Significantly enriched hallmarks (one-sided hypergeometric test, Benjamini-Hochberg multiple testing correction threshold of 5 %) for each cluster are indicated by the presence of circles. The size and colour of the circles represent the number of proteins contributing to that term and the adjusted p value of the enrichment, respectively. Cluster 3 did not show significant pathway enrichment. Source data are provided as a Source Data file.

A

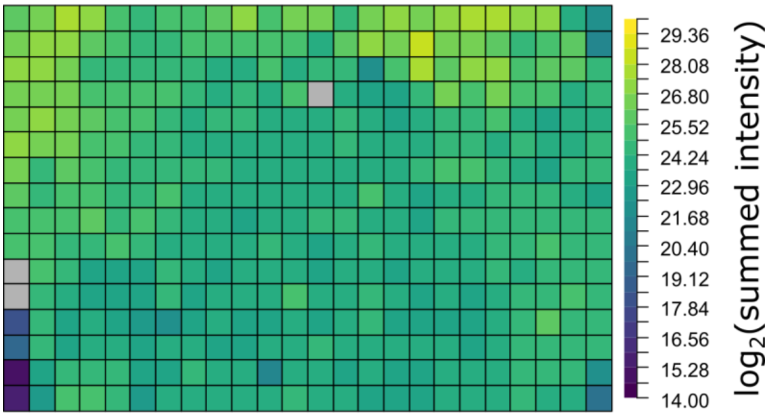

B

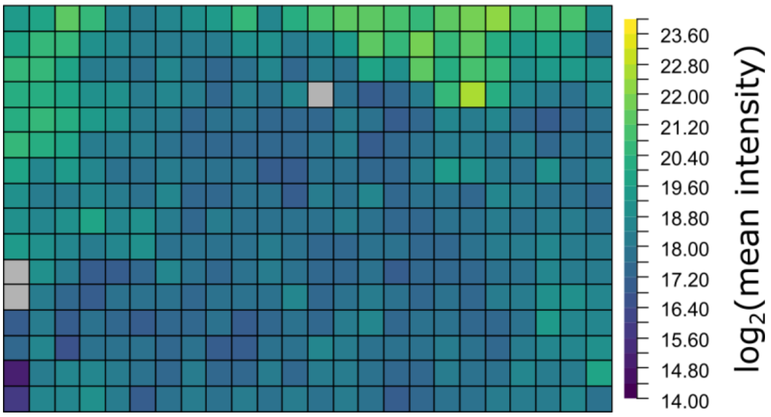

Figure S9 – Aggregate intensity distribution of core matrisome proteins at 833 µm spatial resolution

Maps of the log_2 transformed (A) summed and (B) mean intensities of core matrisome proteins as defined by MatrisomeDB for each voxel. Grey voxels indicate no core matrisome proteins were detected.

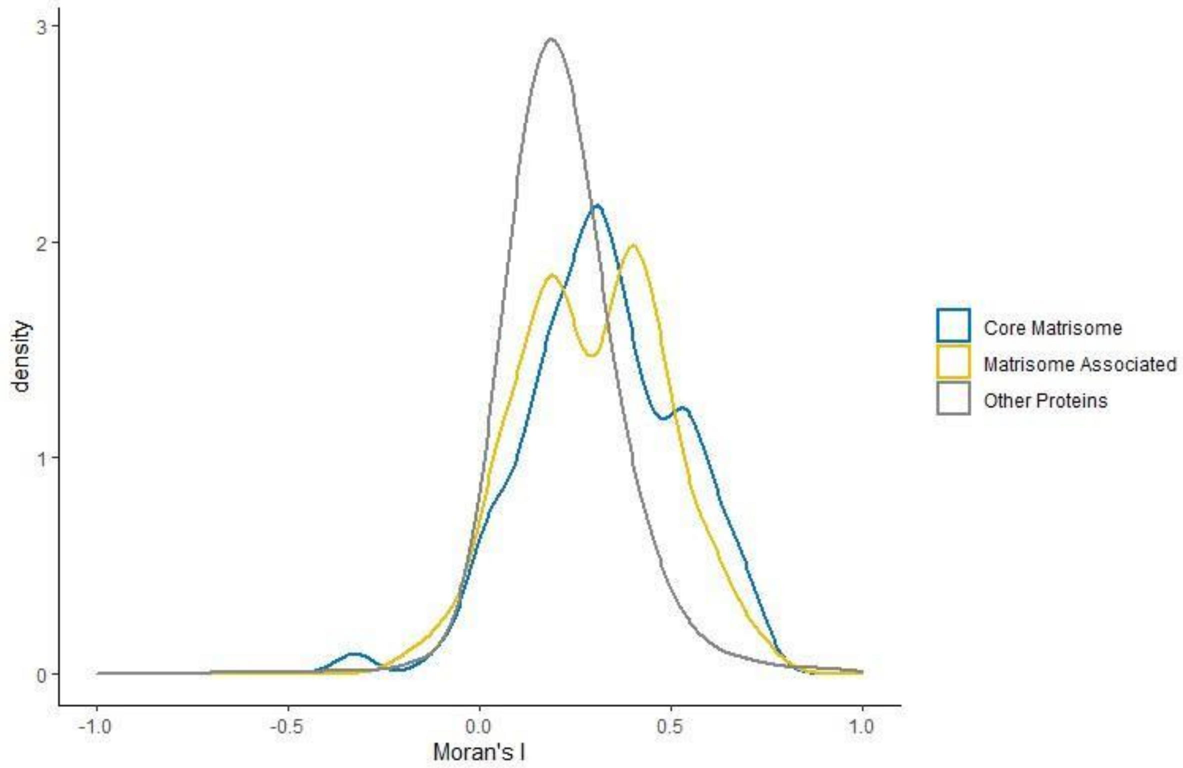

Figure S10 – Distribution of Moran's I in matrisome and non-matrisome proteins 833 µm spatial resolution
Moran's *I* distribution of proteins annotated in Matrisome DB as core matrisome (blue), matrisome associated (yellow), and all other proteins (grey). Source data are provided as a Source Data file.
