# Supplementary material for: Deep topographic proteomics of a human brain tumour
Source: Nat Commun. 2023 Nov 24;14:7710. doi: 10.1038/s41467-023-43520-8 (PMC10673928; doi:10.1038/s41467-023-43520-8)
Supplement: Supplementary file 3 — Description of Additional Supplementary Files [file 41467_2023_43520_MOESM3_ESM.pdf]

## Description of Additional Supplementary Files

**Supplementary Data 1:** Protein identification output of MaxQuant for the 833 µm resolution data

**Supplementary Data 2:** Protein identification output of MaxQuant for the 350 µm resolution data

**Supplementary Data 3:** Protein identification output of DIA-NN for the 40 µm resolution data DIA-

**Supplementary Data 4:** Maps showing the position within the voxel grids for each mass

spectrometry raw file

**Supplementary Data 5:** Whole-slide images of IHC images presented in Supplementary Figure 5
