# Supplementary figures and images for: Deep topographic proteomics of a human brain tumour
Source: Nat Commun. 2023 Nov 24;14:7710. doi: 10.1038/s41467-023-43520-8 (PMC10673928; doi:10.1038/s41467-023-43520-8)

## Slide 1
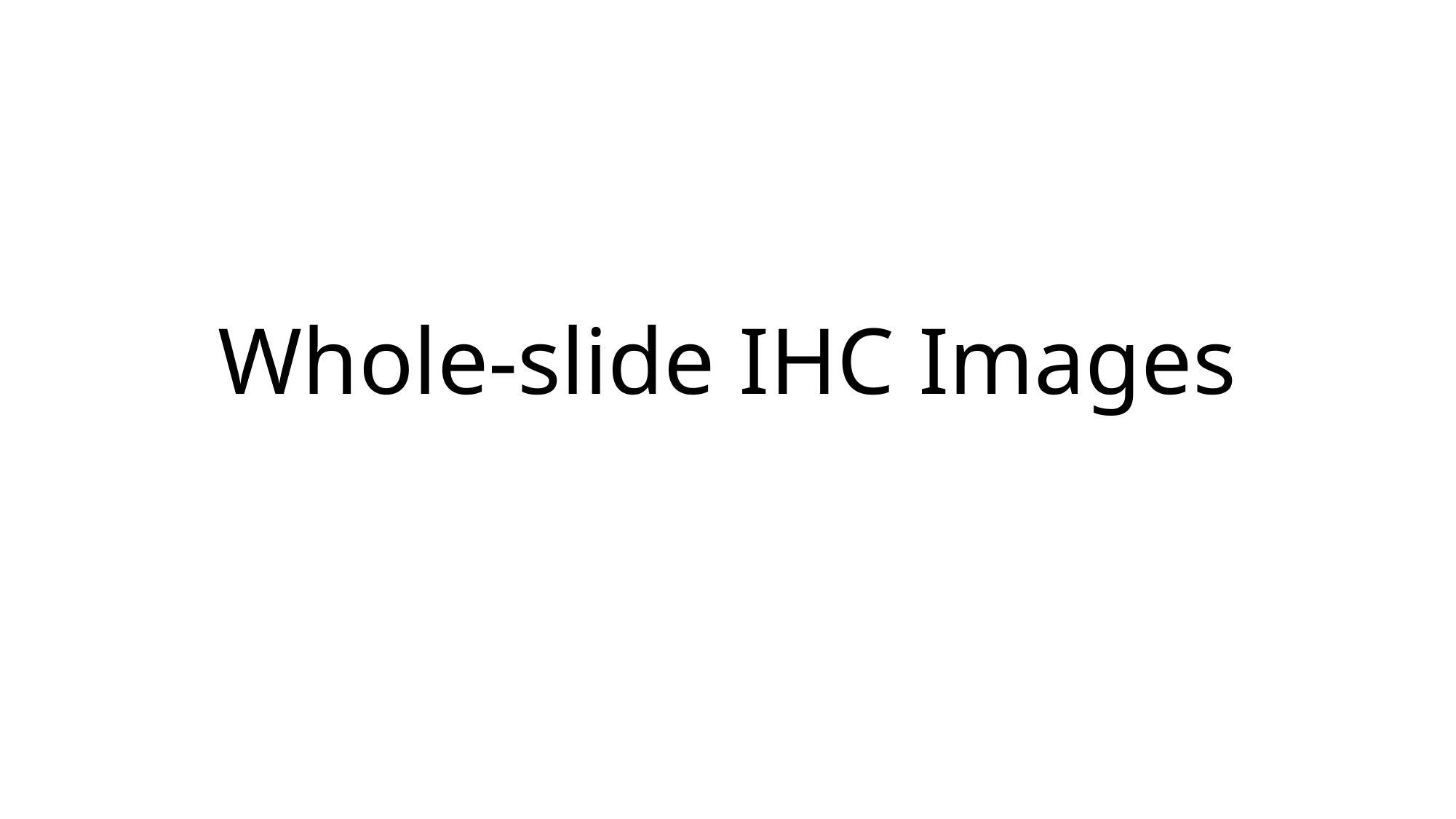

# Whole-slide IHC Images

## Slide 2
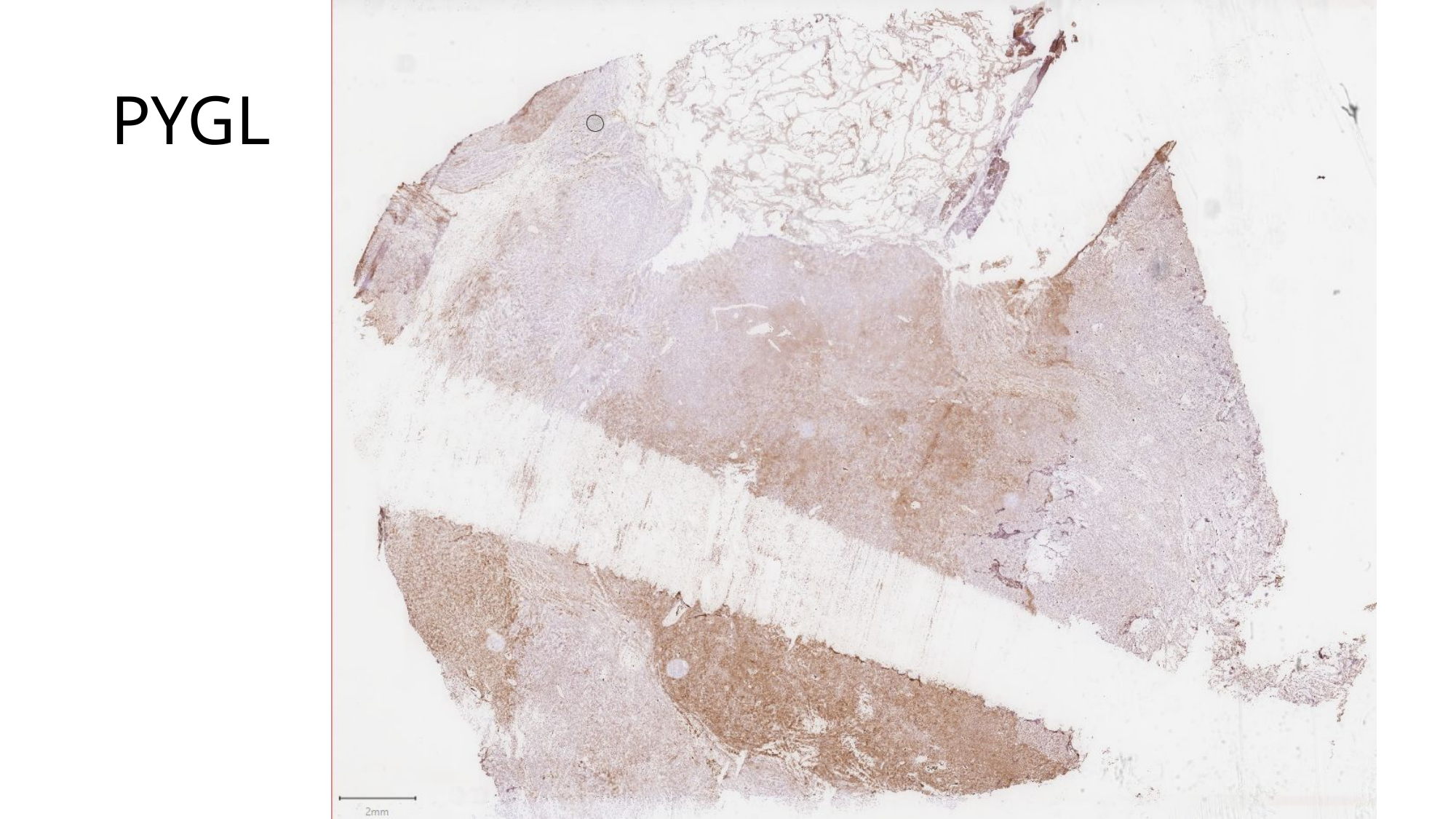

# PYGL

## Slide 3
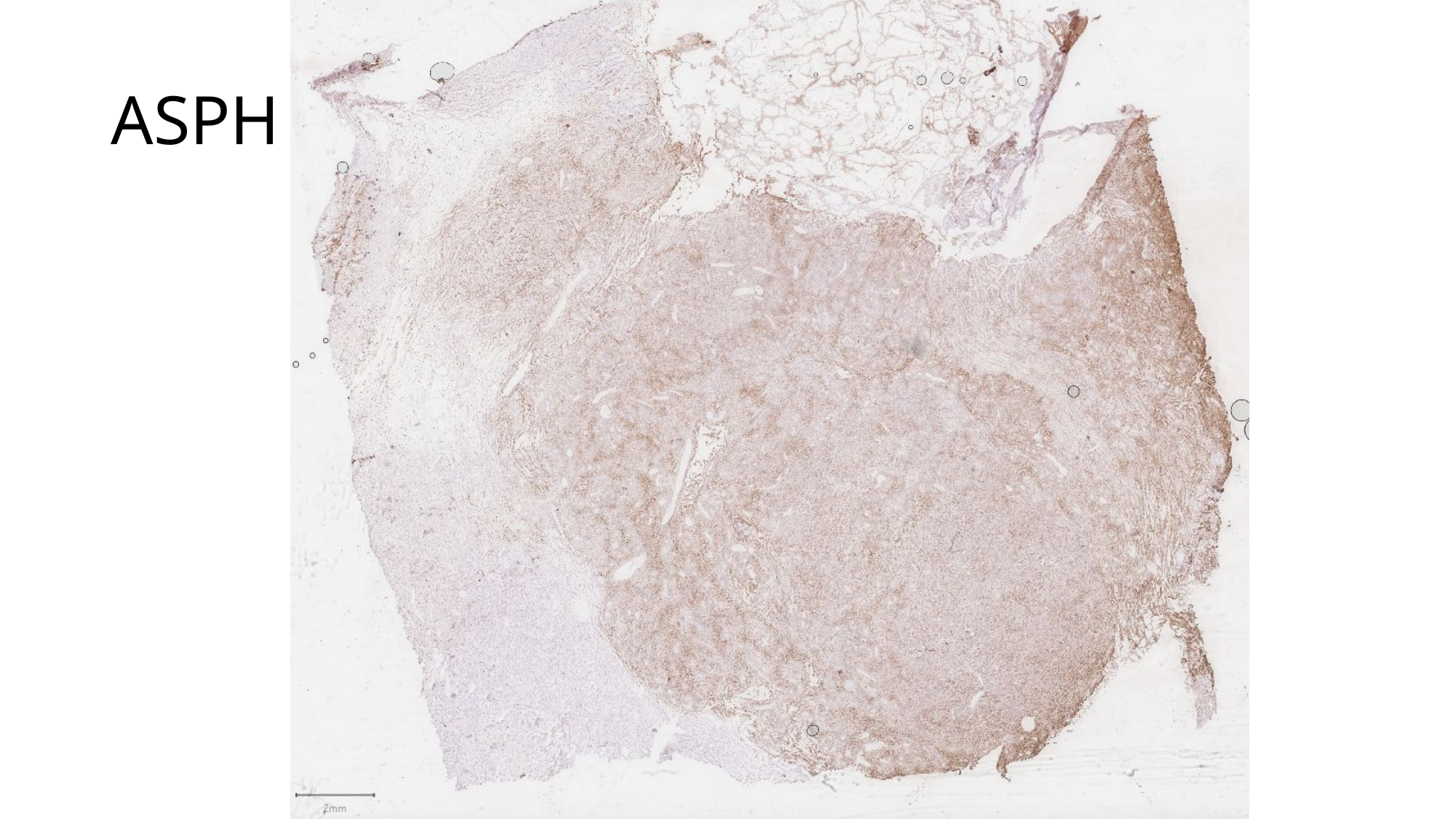

# ASPH

## Slide 4
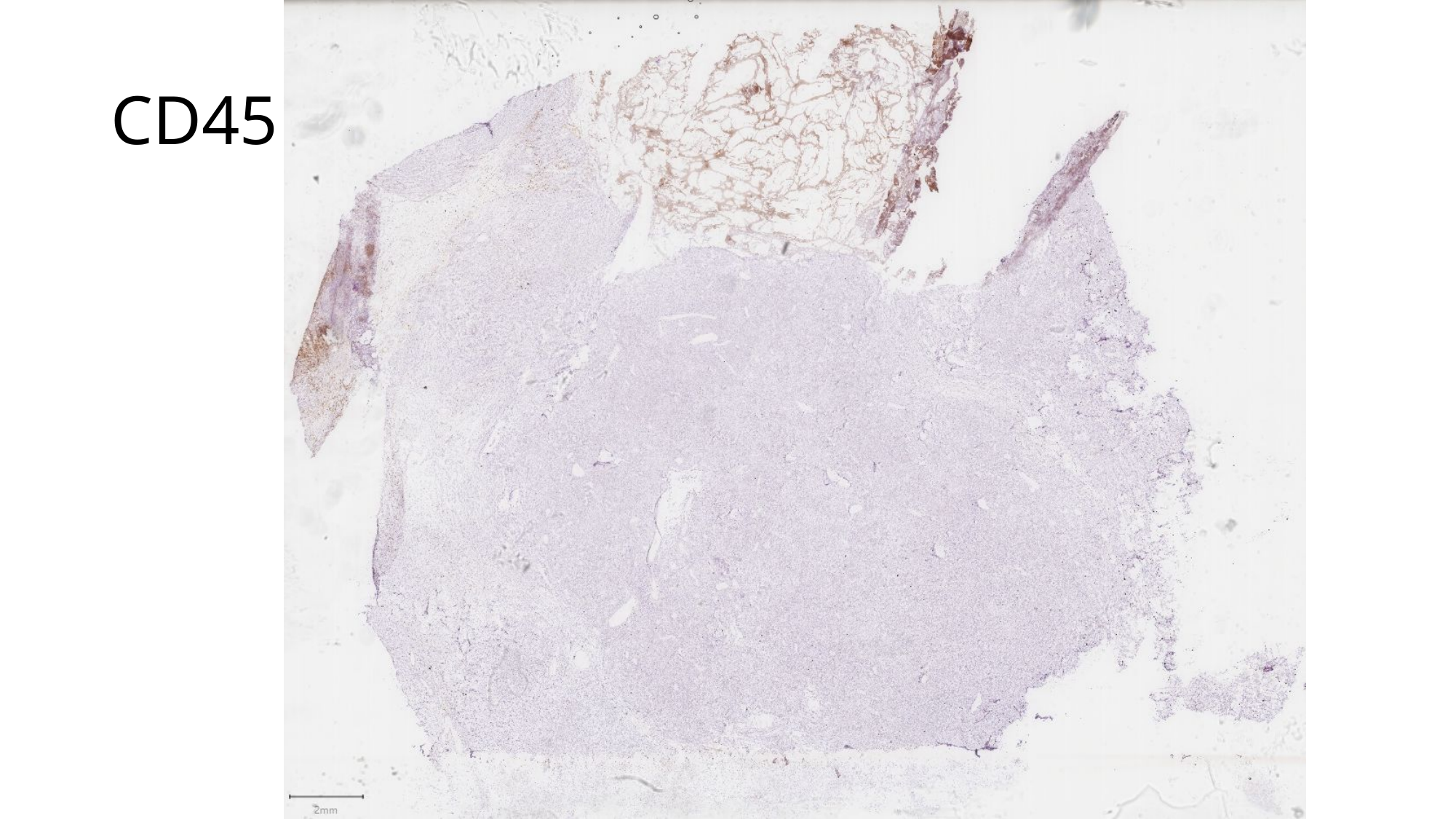

# CD45

Supplement: Supplementary file 8 — Supplementary Data 5 [file 41467_2023_43520_MOESM8_ESM.zip › Whole-slide IHC Images.pptx]
