# Supplementary material for: Deep topographic proteomics of a human brain tumour
Source: Nat Commun. 2023 Nov 24;14:7710. doi: 10.1038/s41467-023-43520-8 (PMC10673928; doi:10.1038/s41467-023-43520-8)
Supplement: Supplementary file 9 — Reporting Summary [file 41467_2023_43520_MOESM9_ESM.pdf]

nature portfolio

Corresponding author(s): Roman Fischer

Last updated by author(s): Oct 10, 2023

Reporting Summary

Nature Portfolio wishes to improve the reproducibility of the work that we publish. This form provides structure for consistency and transparency in reporting. For further information on Nature Portfolio policies, see our Editorial Policies and the Editorial Policy Checklist.

Statistics

For all statistical analyses, confirm that the following items are present in the figure legend, table legend, main text, or Methods section.

n/a	Confirmed	
☐	☒	The exact sample size (*n*) for each experimental group/condition, given as a discrete number and unit of measurement
☐	☒	A statement on whether measurements were taken from distinct samples or whether the same sample was measured repeatedly
☐	☒	The statistical test(s) used AND whether they are one- or two-sided<br>*Only common tests should be described solely by name; describe more complex techniques in the Methods section.*
☒	☐	A description of all covariates tested
☐	☒	A description of any assumptions or corrections, such as tests of normality and adjustment for multiple comparisons
☐	☒	A full description of the statistical parameters including central tendency (e.g. means) or other basic estimates (e.g. regression coefficient) AND variation (e.g. standard deviation) or associated estimates of uncertainty (e.g. confidence intervals)
☐	☒	For null hypothesis testing, the test statistic (e.g. *F*, *t*, *r*) with confidence intervals, effect sizes, degrees of freedom and *P* value noted<br>*Give P values as exact values whenever suitable.*
☒	☐	For Bayesian analysis, information on the choice of priors and Markov chain Monte Carlo settings
☒	☐	For hierarchical and complex designs, identification of the appropriate level for tests and full reporting of outcomes
☐	☒	Estimates of effect sizes (e.g. Cohen's *d*, Pearson's *r*), indicating how they were calculated

Our web collection on statistics for biologists contains articles on many of the points above.

Software and code

Policy information about availability of computer code

Data collection	Zeiss PALM MicroBeam Laser Capture Microscope: PALMRobo v4.5<br>Leica LMD7: Leica LMD v8.2; LAS X v3.7.5<br>QuPath: v0.3.2<br>timsTOF Pro: Hystar 5.1.8.1; oTOFControl 6.2.105<br>timsTOF SCP: Hystar 6.2.1.13; timsControl 4.1.12.0<br>Orbitrap Fusion Lumos: Tune 3.3.2782.23
Data analysis	Data analysis was performed in the R statistical environment (Version 4.0.2). All libraries used are available for download on CRAN (httpss://cran.r-project.org/. Custom code is provided at:<br>https://github.com/pdcharles/spatial-proteomics-anf<br>https://zenodo.org/record/8341909<br><br>Tested on R4.3.1 on Windows 10 (details below)<br>> sessionInfo()<br>R version 4.3.1 (2023-06-16 ucrt)<br>Platform: x86_64-w64-mingw32/x64 (64-bit)<br>Running under: Windows 10 x64 (build 19042)<br><br>Matrix products: default

```
locale:
[1] LC_COLLATE=English_United Kingdom.utf8  LC_CTYPE=English_United Kingdom.utf8
[3] LC_MONETARY=English_United Kingdom.utf8 LC_NUMERIC=C
[5] LC_TIME=English_United Kingdom.utf8

time zone: Europe/London
tzcode source: internal

attached base packages:
[1] parallel  stats     graphics  grDevices utils     datasets  methods   base

other attached packages:
 [1] reshape2_1.4.4       knitr_1.44           ggplot2_3.4.3        RColorBrewer_1.1-3   dynamicTreeCut_1.63-1
 [6] data.table_1.14.8    plot.matrix_1.6.2    viridis_0.6.4        viridisLite_0.4.2    scales_1.2.1
[11] doParallel_1.0.17    iterators_1.0.14     foreach_1.5.2        raster_3.6-23        sp_2.0-0
[16] spdep_1.2-8          sf_1.0-14            spData_2.3.0         dplyr_1.1.3          pacman_0.5.1

loaded via a namespace (and not attached):
 [1] s2_1.1.4           utf8_1.2.3         generics_0.1.3     class_7.3-22       KernSmooth_2.23-21 stringi_1.7.12
 [7] lattice_0.21-8     magrittr_2.0.3     grid_4.3.1         plyr_1.8.8         e1071_1.7-13       DBI_1.1.3
[13] gridExtra_2.3      fansi_1.0.4        codetools_0.2-19   cli_3.6.1          rlang_1.1.1        units_0.8-3
[19] munsell_0.5.0      withr_2.5.0        tools_4.3.1        deldir_1.0-9       colorspace_2.1-0   boot_1.3-28.1
[25] vctrs_0.6.3        R6_2.5.1           proxy_0.4-27       lifecycle_1.0.3    classInt_0.4-10    stringr_1.5.0
[31] pkgconfig_2.0.3    terra_1.7-46       pillar_1.9.0       gtable_0.3.4       glue_1.6.2         Rcpp_1.0.11
[37] xfun_0.40          tibble_3.2.1       tidyselect_1.2.0   farver_2.1.1       labeling_0.4.3     wk_0.8.0
[43] compiler_4.3.1

ANF custom code:
Tested on R version 4.3.0 on macOS Monterey 12.6.5
Directly attached package versions:
magick - 2.7.5
Spectrum - 1.1
ANF - 1.22.0
data.table - 1.14.8
```

For manuscripts utilizing custom algorithms or software that are central to the research but not yet described in published literature, software must be made available to editors and reviewers. We strongly encourage code deposition in a community repository (e.g. GitHub). See the Nature Portfolio guidelines for submitting code & software for further information.

Data

Policy information about availability of data

All manuscripts must include a data availability statement. This statement should provide the following information, where applicable:

- Accession codes, unique identifiers, or web links for publicly available datasets
- A description of any restrictions on data availability
- For clinical datasets or third party data, please ensure that the statement adheres to our policy

The mass spectrometry proteomics data have been deposited to ProteomeXchange Consortium via the PRIDE partner repository. The 833 µm resolution data can be found at PXD039159. The 350 µm resolution data can be found at PXD039398. The 40 µm resolution data can be found at PXD044714.

Research involving human participants, their data, or biological material

Policy information about studies with human participants or human data. See also policy information about sex, gender (identity/presentation), and sexual orientation and race, ethnicity and racism.

Reporting on sex and gender	N/A - Case study on one tissue block, Sex and Gender were not considered when choosing the sample
Reporting on race, ethnicity, or other socially relevant groupings	N/A - None of these characteristics where used when choosing the sample
Population characteristics	Subject age 3.5 years at presentation, disease duration 2.5 years
Recruitment	Case study on one tissue block from the Oxford Brain Bank, block was selected based on a large amount of tumour tissue available for optimisation and follow up work.
Ethics oversight	Post-mortem brain tissue was retrieved by the Oxford Brain Bank; a research ethics committee (REC) approved and Human Tissue Authority (HTA) -regulated research tissue bank (REC reference 15/SC/0639, issued by the NHS Health Research Authority 'South-Central – Oxford C'). Tissue was donated and analysed after full written consent was obtained from the

next of kin of the tissue donor. The Oxford Brain Bank is hosted by the Nuffield Department of Clinical Neurosciences, University of Oxford.

Note that full information on the approval of the study protocol must also be provided in the manuscript.

Field-specific reporting

Please select the one below that is the best fit for your research. If you are not sure, read the appropriate sections before making your selection.

☒ Life sciences ☐ Behavioural & social sciences ☐ Ecological, evolutionary & environmental sciences

For a reference copy of the document with all sections, see nature.com/documents/nr-reporting-summary-flat.pdf

Life sciences study design

All studies must disclose on these points even when the disclosure is negative.

Sample size	No sample size calculations were performed. As this study focuses on methodology, replicate analyses were not performed on additional tumour samples from additional subjects. The number of voxels per experiment were chosen with consideration on MS time (maximum 10 days), spatial coverage of region of interest, and achievable proteomic depth.
Data exclusions	No data were excluded
Replication	We performed spatial analysis of serial tissue sections at three length scales. The results of the two larger length scales broadly agree (the smallest length scale fits entirely within one sampled sub-region of the middle length scale, and so no spatial comparison can be done). The middle length scale experiment was replicated a further two times for practice and testing purposes.
Randomization	Randomisation was not required as this study only involved material from one tissue block
Blinding	Investigators were not blinded during experimentation or analysis due to the study design of working on one tissue block.

Reporting for specific materials, systems and methods

We require information from authors about some types of materials, experimental systems and methods used in many studies. Here, indicate whether each material, system or method listed is relevant to your study. If you are not sure if a list item applies to your research, read the appropriate section before selecting a response.

Materials & experimental systems

n/a	Involved in the study	
☐	☒	Antibodies
☒	☐	Eukaryotic cell lines
☒	☐	Palaeontology and archaeology
☒	☐	Animals and other organisms
☒	☐	Clinical data
☒	☐	Dual use research of concern
☒	☐	Plants

Methods

n/a	Involved in the study	
☒	☐	ChIP-seq
☒	☐	Flow cytometry
☒	☐	MRI-based neuroimaging

Antibodies

Antibodies used	Primary antibodies used and dilutions: rabbit anti-PYGL, 1:100, 4 °C overnight, HPA000962 (Atlas Antibodies); rabbit anti-ASPH, 1:1000, RT 60 minutes, NBP2-34125 (Novus Biologicals); mouse anti-CD45 (PD7/26 + 2B11), 1:200, RT 60 minutes, ab781 (Abcam).
Validation	Anti-PYGL: The manufacturer's website states that this antibody has been tested for WB, IHC, and ICC-IF, and has species reactivity for human. The manufacturer's validation information is as follows:<br><br>Immunohistochemistry (IHC)<br>Orthogonal validation of protein expression using IHC by comparison to RNA-seq data of the corresponding target in high and low expression tissues.<br>Validation of protein expression in IHC by comparing independent antibodies targeting different epitopes of the protein.<br>Validated against independent antibody Anti-PYGL HPA004119.<br>Anti-ASPH: The manufacturer's website states that this antibody has been tested for IHC and has species reactivity for human. The manufacturer's website does not provide a statement on validation, only showing an image of strong astrocyte positivity.<br><br>Anti-CD45: The manufacturer's website states that this antibody has been tested for IHC and has species reactivity for human. The manufacturer's website does not provide a statement on validation, only showing an image of strong lymphocyte staining in tonsil.

Plants

Seed stocks	NA
Novel plant genotypes	NA
Authentication	NA
